# Tuberculosis — United States, 2023

**DOI:** 10.15585/mmwr.mm7312a4

**Published:** 2024-03-28

**Authors:** Paula M. Williams, Robert H. Pratt, William L. Walker, Sandy F. Price, Rebekah J. Stewart, Pei-Jean I. Feng

**Affiliations:** ^1^Epidemic Intelligence Service, CDC; ^2^Division of Tuberculosis Elimination, National Center for HIV, Viral Hepatitis, STD, and TB Prevention, CDC.

**Summary**

**What is already known about this topic?**

For years, the United States has had one of the lowest tuberculosis (TB) rates in the world. In the first year of the COVID-19 pandemic, reported TB case counts dropped substantially, followed by increasing case counts every year since 2020.

**What is added by this report?**

During 2023, tuberculosis case counts increased among all age groups, among U.S-born and non-U.S.–born persons, and in most reporting jurisdictions. Overall, cases increased from 8,320 in 2022 to 9,615 in 2023, an increase of 1,295 cases. The rate also increased from 2.5 per 100,000 persons in 2022 to 2.9 in 2023.

**What are the implications for public health practice?**

Continued progress toward TB elimination will require strong public health systems that are capable of maintaining essential disease prevention and control activities and prepared to withstand the next pandemic or other large-scale crisis.

## Abstract

After 27 years of declining U.S. tuberculosis (TB) case counts, the number of TB cases declined considerably in 2020, coinciding with the COVID-19 pandemic. For this analysis, TB case counts were obtained from the National TB Surveillance System. U.S. Census Bureau population estimates were used to calculate rates overall, by jurisdiction, birth origin, race and ethnicity, and age group. Since 2020, TB case counts and rates have increased each year. During 2023, a total of 9,615 TB cases were provisionally reported by the 50 U.S. states and the District of Columbia (DC), representing an increase of 1,295 cases (16%) as compared with 2022. The rate in 2023 (2.9 per 100,000 persons) also increased compared with that in 2022 (2.5). Forty states and DC reported increases in 2023 in both case counts and rates. National case counts increased among all age groups and among both U.S.-born and non-U.S.–born persons. Although TB incidence in the United States is among the lowest in the world and most U.S. residents are at minimal risk, TB continues to cause substantial global morbidity and mortality. This postpandemic increase in U.S. cases highlights the importance of continuing to engage communities with higher TB rates and their medical providers in TB elimination efforts and strengthening the capacity in public health programs to carry out critical disease control and prevention strategies.

## Introduction

Despite being both preventable and curable, tuberculosis (TB) remains one of the world’s leading infectious disease killers (*1*). The United States has one of the lowest TB rates globally (*1*) and has a goal of eliminating TB (elimination defined as less than one case per 1 million population) by 2035 (*2*). During 1995–2014, health departments and CDC TB control efforts prevented as many as 300,000 persons from developing TB disease and averted up to $14.5 billion in costs (*3*). After 27 years of declining U.S. TB cases, the number of TB cases declined considerably in 2020 to 7,171, coinciding with the COVID-19 pandemic (*4*); however, TB case counts and rates increased in 2021 and 2022. This report provides provisional TB surveillance data for 2023 in the United States. 

## Methods

### Tuberculosis Case Counts and Incidence

The 50 U.S. states and DC report each TB case that meets the Council of State and Territorial Epidemiologists’ surveillance case definition* to CDC’s National Tuberculosis Surveillance System (NTSS).^†^ National case counts, along with counts by jurisdiction, birth origin,^§^ race and ethnicity, and age group, were obtained from NTSS. National and jurisdictional TB rates per 100,000 persons were calculated using the midyear U.S. Census Bureau population estimates,^¶^ and rates by birth origin (i.e., U.S.-born versus non-U.S.–born), race and ethnicity, and age group were calculated using the Current Population Survey** midyear estimates. Percentage changes in TB case counts and rates for 2023 compared with 2022 were calculated overall and by jurisdiction and demographic characteristics. Annual number and rate of TB cases are reported by birth origin for 2013 through 2023. SAS software (version 9.4; SAS Institute) was used for all analyses. This activity was reviewed by CDC, deemed not research, and was conducted consistent with applicable federal law and CDC policy.^††^

### Population Characteristics

Self-reported race and ethnicity were categorized according to federal guidelines.^§§^ Persons of Hispanic or Latino (Hispanic) origin might be of any race but are categorized as Hispanic; all racial groups are non-Hispanic. Non-Hispanic persons who reported more than one race were categorized as “multiple race.” 

## Results

### Tuberculosis Incidence by Jurisdiction

In 2023, the 50 U.S. states and DC provisionally reported 9,615 TB cases, an increase of 1,295 cases (16%) compared with the 8,320 cases reported in 2022, an 8% increase compared with the 2019 prepandemic case count (8,895), and the highest number of cases reported since 2013 (9,556) (Figure). Overall, the U.S. TB rate increased by 15%, from 2.5 per 100,000 persons in 2022 to 2.9 in 2023 (Table 1). Forty states and DC reported an increase in both case counts and rates compared with those in 2022. As in 2022, California reported the highest number of cases in 2023 (2,113), and Alaska reported the highest rate (10.6). Eight states and DC reported TB rates higher than the national rate of 2.9 per 100,000 in 2023.

**FIGURE:**
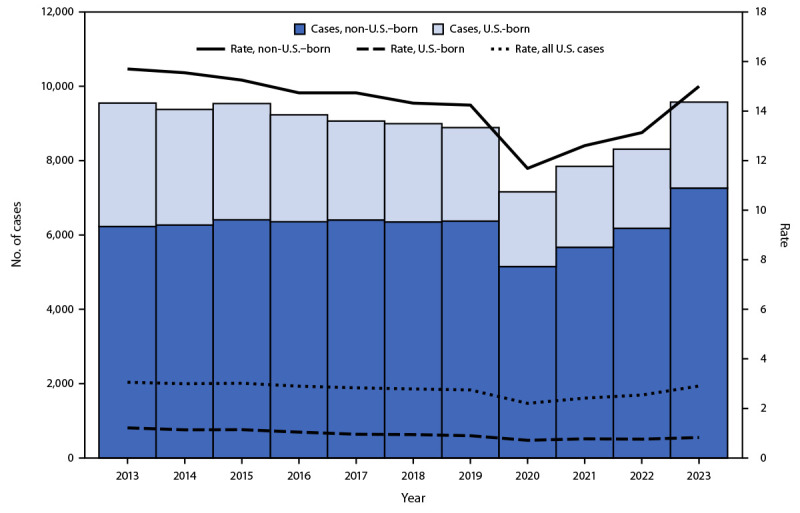
Annual number* and rate^†^ of cases of tuberculosis disease, by birth origin^§^ — United States, 2013–2023 * Case counts are based on data from the National Tuberculosis Surveillance System as of February 17, 2024. ^†^ Annual tuberculosis rate is calculated as cases per 100,000 persons. The Current Population Survey provides the population denominators used to calculate tuberculosis rate according to birth origin. https://www.census.gov/programs-surveys/cps.html (Accessed February 2, 2024). ^§^ Persons born in the United States or certain U.S. territories or elsewhere to at least one U.S. citizen parent are categorized as U.S.-born. All other persons are categorized as non–U.S.–-born. Persons for whom birth origin was unknown (range = 7 [2013] to 42 [2023]) are not included in this figure.

**TABLE 1:** Tuberculosis case counts and rate, by jurisdiction — United States, 2022 and 2023

Jurisdiction	No. of cases*		% Change 2022 to 2023^§^	TB rate^†^		% Change 2022 to 2023^§^
	2022	2023		2022	2023	
**All**	**8,320**	**9,615**	16	**2.5**	**2.9**	15
Alabama	65	92	42	1.3	1.8	41
Alaska	95	78	−18	13	10.6	−18
Arizona	154	202	31	2.1	2.7	30
Arkansas	68	83	22	2.2	2.7	21
California	1,842	2,113	15	4.7	5.4	15
Colorado	57	89	56	1.0	1.5	55
Connecticut	67	66	−1	1.9	1.8	−2
Delaware	13	21	62	1.3	2.0	60
District of Columbia	15	26	73	2.2	3.8	71
Florida	535	624	17	2.4	2.8	15
Georgia	261	248	−5	2.4	2.2	−6
Hawaii	100	116	16	6.9	8.1	16
Idaho	11	15	36	0.6	0.8	35
Illinois	298	353	18	2.4	2.8	19
Indiana	99	130	31	1.4	1.9	31
Iowa	60	67	12	1.9	2.1	11
Kansas	52	46	−12	1.8	1.6	−12
Kentucky	70	75	7	1.6	1.7	7
Louisiana	95	97	2	2.1	2.1	2
Maine	17	26	53	1.2	1.9	52
Maryland	157	198	26	2.5	3.2	26
Massachusetts	154	224	45	2.2	3.2	45
Michigan	120	149	24	1.2	1.5	24
Minnesota	132	160	21	2.3	2.8	21
Mississippi	53	41	−23	1.8	1.4	−23
Missouri	71	72	1	1.1	1.2	1
Montana	6	8	33	0.5	0.7	32
Nebraska	29	33	14	1.5	1.7	13
Nevada	62	86	39	2.0	2.7	38
New Hampshire	11	14	27	0.8	1.0	27
New Jersey	289	330	14	3.1	3.6	14
New Mexico	30	41	37	1.4	1.9	37
New York	709	894	26	3.6	4.6	27
North Carolina	164	215	31	1.5	2.0	29
North Dakota	10	9	−10	1.3	1.1	−11
Ohio	146	193	32	1.2	1.6	32
Oklahoma	77	66	−14	1.9	1.6	−15
Oregon	73	78	7	1.7	1.8	7
Pennsylvania	173	216	25	1.3	1.7	25
Rhode Island	17	27	59	1.6	2.5	59
South Carolina	101	90	−11	1.9	1.7	−12
South Dakota	10	14	40	1.1	1.5	39
Tennessee	106	118	11	1.5	1.7	10
Texas	1,100	1,235	12	3.7	4.0	11
Utah	33	34	3	1.0	1.0	2
Vermont	3	3	0	0.5	0.5	0
Virginia	195	207	6	2.2	2.4	6
Washington	251	222	−12	3.2	2.8	−12
West Virginia	11	15	36	0.6	0.8	37
Wisconsin	52	54	4	0.9	0.9	3
Wyoming	1	2	100	0.2	0.3	99

* Case counts are based on data reported to the National Tuberculosis Surveillance System as of February 17, 2024.

^†^ Annual tuberculosis rate is calculated as cases per 100,000 persons using midyear population estimates from the U.S. Census Bureau. Short-term projections from the monthly population estimates by age, sex, and race and ethnicity were used for the 2023 population. Vintage 2022 estimates were used for 2022 and 2023. https://www.census.gov/programs-surveys/popest/data/tables.html

^§^ Percentage change in rate was calculated with unrounded numbers.

### Tuberculosis Incidence by Demographic Characteristics

In 2023, among 9,573 TB cases in persons for whom birth origin was known, 7,259 (76%) occurred among non-U.S.–born persons, an 18% increase compared with the 6,177 such cases reported in 2022 (Table 2). The number of cases in U.S.-born persons in 2023 increased 9%, from 2,131 in 2022 to 2,314.^¶¶^ The rate increased among non-U.S.–born persons from 13.1 in 2022 to 15.0 in 2023, and the rate among U.S.-born persons remained at 0.8 cases per 100,000 persons.

**TABLE 2:** Characteristics of persons with tuberculosis — United States, 2022 and 2023

Characteristic	No. of cases* (%)		% Change 2022 to 2023^§^	TB rate^†^		% Change 2022 to 2023^§^
	2022	2023		2022	2023	
**Overall**	**8,320**	**9,615**	**16**	**2.5**	**2.9**	**15**
**Age group,^¶^** **yrs**						
0–4	199 (2)	233 (2)	17	1.1	1.3	17
5–14	163 (2)	231 (2)	42	0.4	0.6	45
15–24	844 (10)	1,017 (11)	21	2.0	2.3	16
25–44	2,450 (29)	3,001 (31)	22	2.8	3.4	21
45–64	2,416 (29)	2,597 (27)	7	2.9	3.2	9
≥65	2,248 (27)	2,530 (26)	13	4.0	4.3	9
**Race and ethnicity**						
U.S.-born**^,††,§§^	2,131 (26)	2,314 (24)	9	0.8	0.8	8
American Indian or Alaska Native	113 (5)	106 (5)	−6	4.5	4.1	−9
Asian	142 (7)	130 (6)	−8	1.7	1.5	−12
Black or African American	672 (32)	753 (33)	12	1.9	2.1	12
Native Hawaiian or other Pacific Islander	51 (2)	62 (3)	22	6.4	7.7	20
White	569 (27)	591 (26)	4	0.3	0.3	4
Hispanic or Latino	542 (25)	614 (27)	13	1.3	1.5	11
Multiple races	20 (1)	18 (1)	−10	0.3	0.2	−14
**Non–U.S.**–born**^,§§,¶¶^	6,177 (74)	7,259 (76)	18	13.1	15.0	14
American Indian or Alaska Native***	0 (—)	6 (1)	—	0.0	12.3	—
Asian	2,739 (44)	2,804 (39)	2	22.9	22.5	−2
Black or African American	650 (11)	922 (13)	42	14.2	18.2	28
Native Hawaiian or other Pacific Islander	105 (2)	115 (2)	10	28.4	36.6	29
White	274 (4)	300 (4)	9	3.4	3.7	10
Hispanic or Latino	2,278 (37)	2,876 (40)	26	10.5	12.9	23
Multiple races	68 (1)	64 (1)	−6	28.3	25.8	−9

**Abbreviation: **TB = tuberculosis.

* Case counts are based on data reported to the National Tuberculosis Surveillance System as of February 17, 2024.

^†^ Annual tuberculosis rate is calculated as cases per 100,000 persons using midyear population estimates from the U.S. Census Bureau. Short-term projections from the monthly population estimates by age, sex, and race and ethnicity were used for the 2023 population. Vintage 2022 estimates were used for 2022 and 2023. https://www.census.gov/programs-surveys/popest/data/tables.html

^§^ Percentage change in rate was calculated with unrounded numbers.

^¶^ Age was missing or unknown for zero cases in 2022 and six cases in 2023.

** Persons born in the United States or certain U.S. territories or elsewhere to at least one U.S. citizen parent are categorized as U.S.-born. All other persons are categorized as non-U.S.–born.

^††^ Birth origin was missing or unknown for 12 cases in 2022 and 42 cases in 2023.

^§§ ^Race and ethnicity was missing or unknown for 22 cases in 2022 and 40 cases in 2023 among U.S.-born persons.

^¶¶^ Race and ethnicity was missing or unknown for 63 cases in 2022 and 172 cases in 2023 among non–U.S.–-born persons.

*** No TB cases reported among American Indian or Alaska Native persons in 2022.

Among U.S.-born persons with TB, 33% (753) identified as Black or African American (Black), 27% (614) as Hispanic, 26% (591) as White, 6% (130) as Asian, 5% (106) as American Indian or Alaska Native, 3% (62) as Native Hawaiian or other Pacific Islander, and 1% (18) as multiple race. Among U.S.-born persons, the rate of TB in 2023 compared with 2022 increased 20% (11 cases) among Native Hawaiian or other Pacific Islander, 12% (81 cases) among Black, 11% (72 cases) among Hispanic, and 4% (22 cases) among White persons, and the rate declined 9% (–7 cases) among American Indian or Alaska Native, and 12% (–12 cases) among Asian persons. Among non-U.S.–born persons with TB, 40% (2,876) identified as Hispanic, 39% (2,804) as Asian, 13% (922) as Black, 4% (300) as White, 2% (115) as Native Hawaiian or other Pacific Islander, 1% (64) as multiple race, and 0.1% (six) as American Indian or Alaska Native persons. Among non-U.S.–born persons, the TB rate in 2023 compared with 2022 increased 29% (10 cases) among Native Hawaiian or other Pacific Islander, 28% (272 cases) among Black, 23% (598 cases) among Hispanic, and 10% (26) among White persons, among non-U.S.–born Asian persons, the rate declined 2% (65 cases).***

TB incidence increased in every age group in 2023 compared with 2022, with the largest relative increase among children aged 5–14 years (68 cases, corresponding to a 42% increase in case count and a 45% increase in rate). Among the 83% (8,013) of persons with TB in 2023 for whom HIV status was known, 5% were coinfected with TB and HIV.

## Discussion

Provisional national surveillance data show that TB case counts and rates have increased since the COVID-19 pandemic, returning to the number of cases last observed in 2013 (*4*). Increases occurred in every age group and all except 10 U.S. states. Case counts increased among both U.S.-born and non-U.S.–born persons, with the most substantial increase, 18%, among non-U.S.–born persons (1,082 cases).

The United States has one of the lowest TB rates in the world (*1*) and most U.S. residents are at minimal risk for TB (*2**,**4*). The overall epidemiology of TB continues to reflect persistent disparities by birth origin, and race and ethnicity in the United States. TB rates in 2023 were highest among non-U.S.–born persons which is consistent with prepandemic trends. Among U.S.-born persons, rates remained <1.0 overall but were highest among those who identified as Native Hawaiian or other Pacific Islander, American Indian or Alaska Native, or Black.

Approximately 85% of TB cases in the United States are attributed to reactivation of latent TB infection (LTBI) rather than recent transmission (*2*,*4*). Therefore, sustained transmission of TB in the United States leading to outbreaks is uncommon. Essential TB elimination activities include TB testing among populations at risk and treating persons with LTBI or TB disease. To prevent transmission and reduce morbidity, TB disease must be detected quickly; effective treatment must be initiated promptly; and all exposed persons identified, evaluated, and treated if infected (*5*). This approach led to a 66% reduction in TB cases and 73% reduction in the TB rate in the United States in the first 25 years of implementation (*4*).

TB prevention and control interventions are primarily conducted by staff members in state and local public health programs. The decades-long downward trend in TB in the United States and the high TB disease treatment completion rates (*4*) underscore the success of these TB programs. However, during the COVID-19 pandemic, TB programs were severely taxed with many staff members and activities diverted to the COVID-19 response (*6*). Timely diagnosis and treatment of TB disease also suffered because of pandemic-related disruptions in health care access and health care workers focusing on identifying persons with COVID-19, who often have symptoms similar to those of pulmonary TB (*7*). These factors, along with changes in migration volume (*8*), probably contributed to the decrease in the number of cases observed in 2020, and to the subsequent rise in case counts and rates since 2020. Identification of TB cases possibly increased after the pandemic because of renewed attention to infectious diseases other than COVID-19.

The number of persons who received a new TB diagnosis has also risen globally. In 2022, the World Health Organization reported a second consecutive year of increasing TB case counts, with the global estimate of TB cases equaling that of 2016 (*1*). TB is not the only preventable communicable disease resurging after the COVID-19 pandemic. For example, influenza (*9*) and measles (*10*) have also experienced postpandemic surges. Setbacks to TB elimination in the United States illustrate the power of pandemics and other large-scale crises to have long-lasting effects on public health, a phenomenon also observed at the onset of the HIV epidemic when the number of TB cases increased after 3 decades of decline (*4*). Renewed progress toward TB elimination will require strengthened capacity of public health programs to carry out critical TB control and prevention strategies and engagement of providers and affected communities in TB elimination efforts. In addition, because most TB cases in the United States occur among non-U.S.–born persons, collaboration of public health entities in the United States with international partners is important to reduce TB morbidity globally.

### Limitations

The findings in this report are subject to at least two limitations. First, this analysis is limited to provisional surveillance data for 2023, and case counts might change before CDC’s annual TB surveillance report is published. Second, rates are based on midyear population estimates from the U.S. Census Bureau that are subject to ongoing refinement.

### Implications for Public Health Practice

The U.S. TB case count increases in 2023 underscores the ongoing global TB-associated morbidity and mortality. Renewed progress toward TB elimination will require strong public health systems both domestically and globally that are responsive to health disparities, capable of maintaining essential disease prevention and control activities, and prepared to withstand the next pandemic or other large-scale crisis.

## Footnotes

*
https://ndc.services.cdc.gov/case-definitions/tuberculosis-2009

† This report is limited to National Tuberculosis Surveillance System data verified as of February 17, 2024. Updated data will be available in CDC’s annual TB surveillance report later in 2024.

§ Persons born in the United States or certain U.S. territories or elsewhere to at least one U.S. citizen parent are categorized as U.S.-born. All other persons are categorized as non-U.S.–born.

¶ Short-term projections from the monthly population estimates by age, sex, and race and ethnicity were used for the 2023 population. Vintage 2022 Estimates were used for 2023 and 2022, and Vintage 2010 Estimates were used for 2013–2019. https://www.census.gov/programs-surveys/popest/data/tables.html

**
https://www.census.gov/programs-surveys/cps.html

†† 45 C.F.R. part 46.102(l)(2), 21 C.F.R. part 56; 42 U.S.C. Sect. 241(d); 5 U.S.C. Sect. 552a; 44 U.S.C. Sect. 3501 et seq.

§§
https://www.census.gov/topics/population/race/about.html

¶¶ Proportions using birth origin are calculated excluding 12 cases in 2022 and 42 cases in 2023 for which birth origin was missing or unknown.

*** Percentage change is calculated from unrounded numbers. For demographic groups with small populations (e.g., non-U.S.–born American Indian or Alaska Native), changes in rates should be interpreted cautiously because of the increased volatility of these rates.
